# Correlation of Plasma pTau‐181/Aβ‐42 Ratio with Cuneus and Precuneus Volumes in Alzheimer's Disease

**DOI:** 10.1002/alz70856_100765

**Published:** 2025-12-24

**Authors:** Pukovisa Prawiroharjo, Amelia Nur Vidyanti, Yuliarni Syafrita, Alya Ayu Tazkia, Viola Aprillia, Dinda Nisrina, Elizabeth Divina, Rani Permata, Talitha Najmillah Sabtiari, Alditya Fakhri, Rifki Habibi, Rahma Atika, Nailatul Fadhilah, Nanda Saripa Putri

**Affiliations:** ^1^ Department of Neurology, Faculty of Medicine, Universitas Indonesia, Jakarta, Indonesia, Central Jakarta, Jakarta, Indonesia; ^2^ Dr. Cipto Mangunkusumo Public Hospital, Central Jakarta, DKI Jakarta, Indonesia; ^3^ Department of Neurology, Universitas Gadjah Mada, Yogyakarta, Indonesia, Yogyakarta, Indonesia; ^4^ Department of Neurology, Universitas Andalas, Sumatera barat, indonesia, Kota Padang, Sumatera Barat, Indonesia; ^5^ Brainz Research and Innovation Center, Jakarta, Indonesia; ^6^ Department of Neurology, Universitas Gadjah Mada, Yogyakarta, Indonesia, Yogyakarta, Yogyakarta, Indonesia

## Abstract

**Background:**

Alzheimer's Disease (AD) is the most common form of dementia, neuropathologically characterized by the accumulation of extracellular amyloid‐beta (Aβ) plaques and intracellular tau tangles in the brain cortex, including the cuneus and precuneus region associated with episodic memory and visuospatial processing. In Indonesia, the prevalence of AD is significantly high, affecting 20‐38% of the elderly population, compared to 4‐11% in other Asian countries. Current diagnostic methods, including cerebrospinal fluid (CSF) biomarkers and PET imaging, are limited due to their high cost, invasive nature, and complexity.

**Method:**

A cross‐sectional study will be conducted, including (*n* = 40) AD patients subjects from three hospitals in Indonesia: Dr. Sardjito Hospital (Yogyakarta), Dr. Cipto Mangunkusumo Hospital (Jakarta), and Dr. M. Djamil Hospital (Padang). Plasma and CSF levels of Aβ42 and pTau‐181 will be measured using enzyme‐linked immunosorbent assay (ELISA). The diagnostic accuracy of plasma biomarkers will be assessed using the Youden index and area under the curve (AUC) from receiver operating characteristic (ROC) analyses. The correlation between plasma pTau‐181/Aβ‐42 ratio and brain volume reductions in the cuneus and precuneus will be examined using volumetric MRI.

**Result:**

The volumetric analysis of the subjects' brains revealed mean values (± standard deviation): Right Cuneus (1.77 ± 0.33), Left Cuneus (1.74 ± 0.31), Right Precuneus (1.92 ± 0.38), and Left Precuneus (1.95 ± 0.34). A Correlation between Plasma pTau‐181/Aβ‐42 ratio towards cuneus and precuneus volume show significant correlations (*p* <0.05) with right, left cuneus and right precuneus but not left cuneus (*p* >0.05) which are regions critically involved in cognitive functions such as visuospatial processing and episodic memory.

**Conclusion:**

The potential of the plasma pTau‐181/Aβ‐42 ratio as a non‐invasive biomarker for Alzheimer's Disease shows significant correlations with volumetric changes in the cuneus and precuneus regions of the brain involved in cognitive function. Therefore, plasma biomarkers could serve as a cost‐effective alternative to CSF biomarkers, addressing challenges of invasiveness and high costs in AD diagnostics, particularly in resource‐limited settings like Indonesia.

